# Phosphorylation of Tau R2 Repeat Destabilizes Its Binding to Microtubules: A Molecular Dynamics Simulation Study

**DOI:** 10.1021/acschemneuro.2c00611

**Published:** 2023-01-20

**Authors:** Viet Hoang Man, Xibing He, Jie Gao, Junmei Wang

**Affiliations:** † Department of Pharmaceutical Sciences and Computational Chemical Genomics Screening Center, School of Pharmacy, University of Pittsburgh, Pittsburgh, Pennsylvania15261, United States; ‡ Department of Neuroscience, The Ohio State University Wexner Medical Center, Columbus, Ohio43210, United States

**Keywords:** phosphorylation, tau protein, R2 repeat, free energy

## Abstract

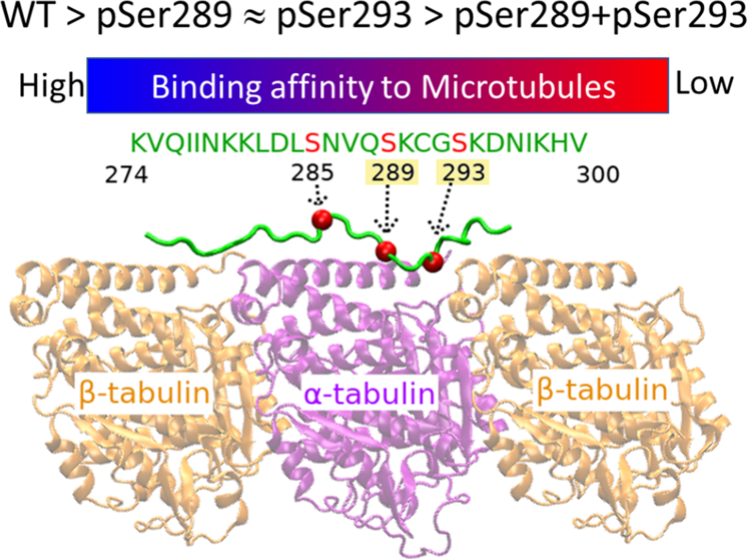

Phosphorylation, the most popular post-translational modification of tau protein, plays an important role in regulating tau physiological functions. However, aberrant phosphorylation attenuates the binding affinity of tau to a microtubule (MT), resulting in MT destabilization followed by accumulation of neurofibrillary tangles in the brain. There are in total 85 potential phosphorylation sites in a full-length tau protein, and about half of them are abnormal as they occur in tau of Alzheimer’s disease (AD) brain only. In this work, we investigated the impact of abnormal Ser289, Ser293, and Ser289/Ser293 phosphorylation on tau R2–MT binding and the conformation of tau R2 using molecular dynamics simulation. We found that the phosphorylation significantly affected R2–MT interaction and reduced the binding affinity of tau R2 peptides to MTs. Free energy decomposition analysis suggested that the post-translational modified residues themselves made a significant contribution to destabilize tau repeat R2–MT binding. Therefore, the phosphorylation may attenuate the binding affinity of tau to MTs. Additionally, the phosphorylation also enhanced helix–coil transition of monomeric R2 peptides, which may result in the acceleration of tau aggregation. Since these phosphorylated sites have not been examined in previous experimental studies, our finding through all-atom molecular dynamics simulations and free energy analysis can inspire experimental scientists to investigate the impact of the phosphorylation on MT binding and aggregation of full-length tau and the pathological roles of the phosphorylation at those sites in AD development through in vitro/in vivo assays.

## Introduction

Microtubules (MTs) are made up of αβ-tubulin dimers and regulated by MT-associated proteins (MAPs). Tau constitutes more than 80% of neuronal MAPs. It stabilizes and bundles axonal MTs. Tau proteins are present as six isoforms with the length ranging from 352 to 441 residues. Full-length adult tau includes an N-terminal projection, a proline-rich domain, an MT-binding region, and a C-terminal domain.^1^ The MT-binding region contains three or four imperfect sequence repeats, R1 to R4. The binding affinity to MTs and the activity of tau increase with the number of repeats.^2,3^ Tau can undergo various post-translational modifications including phosphorylation, acetylation, glycosylation, methylation, nitration, sumoylation, truncation, and ubiquitination.^4,5^ Among these modifications, phosphorylation is of great interest and has been most extensively studied. Tau phosphorylation plays both physiological and pathological roles. The phosphorylation of tau increases during several physiological processes including development, hibernation, and hypothermia.^6−8^ In a healthy brain, tau regulates MTs through a phosphorylated/dephosphorylated reversible process. In the development of neurodegenerative tauopathies, such as Alzheimer’s disease (AD), abnormally phosphorylated tau loses affinity for MTs and forms neurofibrillary tangles (NFTs).^9^

The full-length adult tau has 85 potential phosphorylation sites including 45 serine, 35 threonine, and 5 tyrosine residues. Of these sites, about 20 residues undergo phosphorylation in tau from both healthy and AD brains, while about 40 phosphorylated residues occur only in AD brain.^10^ The different phosphorylation sites and their combinations (namely, multi-site phosphorylation) have different effects on tau aggregation and MT binding affinity. Tau phosphorylation at Thr175 leads to fibril formation and enhances cell death.^11^ Pseudo-phosphorylation of tau at Thr212,^12^ Ser202, and Thr205^13^ promotes tau filament formation. Ser422 pseudo-phosphorylation increases tau oligomerization.^14^ Triple phosphorylation at Ser202/Thr205/Ser208 can lead to a rapid tau aggregation,^15^ while some phosphorylation sites such as Ser214, Ser262, and Ser305 inhibit tau aggregation.^16,17^ Phosphorylation at Ser231 or Ser262 can inhibit tau–MT interaction.^18,19^ In contrast, phosphorylation at Ser208 enhances the binding affinity of tau to MTs.^20^ Despite numerous efforts made to understand the impact of phosphorylation sites on the development of tauopathies in terms of tau–MT interaction and tau aggregation, the effect of many phosphorylation sites is still elusive.

The structure of full-length tau has not been solved by current experimental methods due to its intrinsically disordered nature. However, the interaction of tau repeats with MTs has been studied using biochemical and biophysical techniques.^21−24^ Using a cryo-EM experiment, Kellogg et al. obtained a high-resolution atomistic model of tau repeat R2 in complex with a tubulin trimer (PDB 6CVN), and they also reconstructed the complex of full-length tau and MTs at a resolution of 4.1 Å (EMDB code 7522).^23^ On the other side, molecular dynamics (MD) simulation, which is complementary to experiment, can provide a detailed picture of the structures and dynamics of a biomolecular system at the atomistic level. MD simulation has been applied to investigate not only the structures and dynamics of tau proteins or tau fragments but also tau repeats interacting with MTs.^25−30^ Tau repeat R2 contains three serine residues, Ser285, Ser289, and Ser293, which are potential phosphorylation sites; however, only phosphorylation at Ser289 and Ser293 was observed in tau protein of AD brain only, and there is no report on the phosphorylation at Ser285 of tau protein. In this work, we study the effect of Ser289, Ser293, and Ser289/Ser293 phosphorylation of tau repeat R2 binding to MT tubulins and its conformations in explicit water molecules using all-atom MD simulation. To the best of our knowledge, how the two abnormal phosphorylation sites affect tau–MT interaction and tau aggregation has not been reported yet.

## Results and Discussion

For the sake of convenience, in the following content, we name the phosphorylated Ser residue pSer. Ser is a neutral residue, while the net charge of pSer is −2. Tau repeat R2 contains three potential phosphorylation sites including Ser285, Ser289, and Ser293. The phosphorylated residues at Ser289 and Ser293 occur only in AD brain, and the phosphorylated residue at Ser285 has not been discovered in either healthy or AD brain.^10^ Therefore, we only considered four forms of R2 monomers: the monomer which is a wild-type (R2-WT); the monomer with pSer289 (R2-pSer289); the monomer with pSer293 (R2-pSer293); and the monomer with pSer289 and pSer293 (R2-pSer289 + pSer293).

### Phosphorylation at Ser289 and Ser293 Decreases Binding Affinity of Tau Repeat R2 to MTs

**Figure 1:**
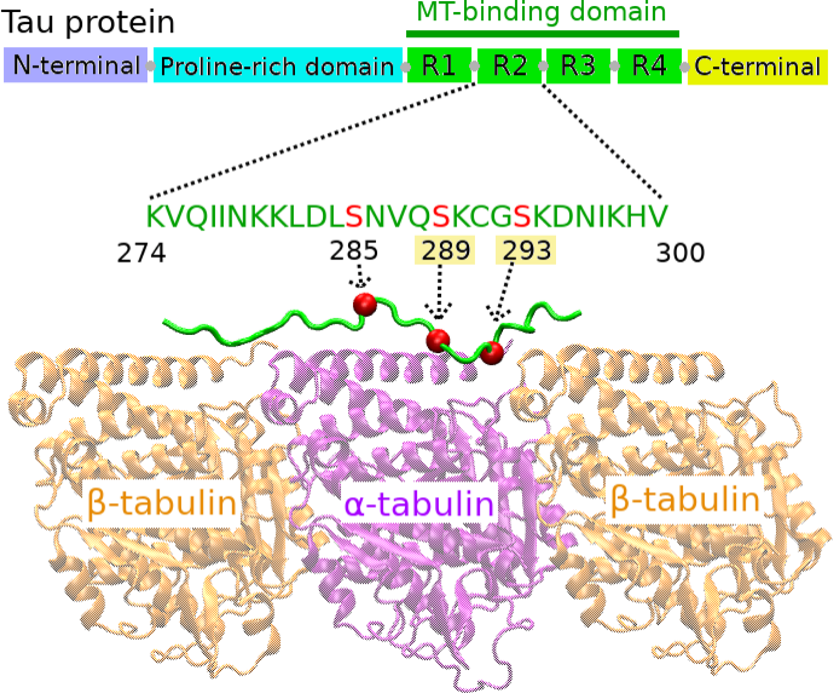
Cryo-EM structure of tau R2 repeat bound to MTs. Among the three serine residues (marked by red balls), phosphorylation at Ser289 and Ser293 was found in AD brain only.

**Figure 2:**
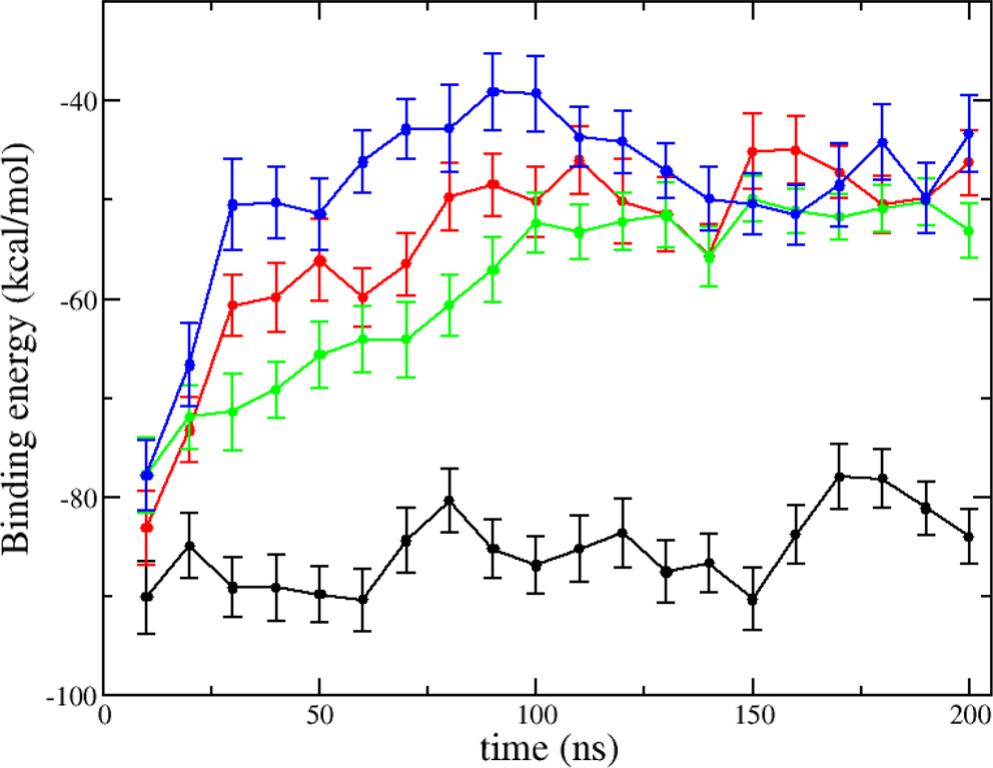
Time courses of the MM-GBSA free energy of tau R2-WT (black), R2-pSer289 (red), R2-pSer293 (green), and R2-pSer289 + pSer293 (blue) peptides binding to MTs. The data were averaged over five independent MD trajectories for each system. Note that the MM-GBSA energies do not include the entropy term.

To examine the binding affinity of tau R2 peptides to MTs, we used an experimental structure of tau R2 peptide binding to MTs, which was recently determined by electron microscopy (Figure 1).^23^ For three phosphorylated R2 systems, we performed computational mutagenesis from Ser to pSer at the corresponding positions of R2-WT. After canonical ensembles were sampled by MD, we then calculated the free energies of the four different R2 peptides binding to MTs using molecular mechanics-generalized Born surface area (MM-GBSA) and weighted solvent accessible surface area (WSAS) methods.^31,32^Figure 2 illustrates the time evolution of MM-GBSA binding energy between an R2 peptide and MT tubulins. Note that the entropy term was not included in MM-GBSA binding free energy calculations. It shows that the binding free energies reach equilibrium after 50 ns for all the four complex systems. The time courses of R2 reaction coordinates including helix content, coil content, solvent-accessible surface area (SASA), and root mean square deviation (RMSD) also fluctuate around equilibrium values after 50 ns (Figure S1 in the Supporting Information). The average values of binding energies calculated using the last 150 ns of the simulations are −84.7 ± 4.5, −50.5 ± 4.9, −55.2 ± 4.7, and −45.9 ± 5.5 kcal/mol for R2-WT, R2-pSer289, R2-pSer293, and R2-pSer289 + pSer293, respectively. The binding free energy and different interaction energy terms of an R2 peptide binding to MT tubulins are shown in Table 1. With the entropy contribution being taken into account, the binding free energies of R2 peptide/MT are −39.9 ± 3.1, −8.7 ± 3.4, −12.3 ± 2.6, and −1.5 ± 3.4 for R2-WT, R2-pSer289, R2-pSer293, and R2-pSer289 + pSer293, respectively. Our result indicated that the phosphorylation at Ser289, Ser293, or Ser289 + Ser293 reduced the affinity of R2 peptides binding to MT tubulins. Using the same model (PDB code 6CVN), MD simulation, and MMPBSA method, Bhandare et al. obtained the binding energy of R2-WT and MT tubulins of about −927.9 ± 3.2 kcal/mol,^25^ which is much lower than our result (−84.7 ± 4.5 kcal/mol). This significant difference is mainly from the differences of electrostatic (Δ*G*_elec_) and polar (Δ*G*_p_^sol^) terms between ours and Bhandare et al.’s results. Note that, our result was calculated from five 150 ns independent MD trajectories, much longer than those sampled from 30 ns MD simulations by Bhandare et al. It is pointed out that the huge difference between the absolute values of the Δ*G*_elec_ and Δ*G*_p_^sol^ terms in Bhandare et al.’s calculation is unusual in MMGB/PBSA calculation.

**Table 1:** Binding Free Energy of Tau R2 Peptides to MT Tubulins^a^

system	Δ*E*_vdw_	Δ*E*_eel_	Δ*G*_p_^sol^	Δ*G*_np_^sol^	*T*Δ*S*	Δ*G*_bind_
R2-WT	–110.3 ± 3.0	–2158.4 ± 26.5	2200.7 ± 26.2	–16.7 ± 0.4	–44.8 ± 0:8	–39.9 ± 3.1
R2-PSer289	–99.0 ± 3.2	–1168.9 ± 24.5	1232.4 ± 23.9	–15.0 ± 0.4	–41.8 ± 0.7	–8.7 ± 3.4
R2-pSer293	–88.7 ± 2.5	–1158.2 ± 21.6	1205.1 ± 21.0	–13.4 ± 0.3	–42.9 ± 0.5	–12.3 ± 2.6
R2-pSer289 + pSer293	–120.2 ± 3.1	–214.2 ± 22.6	306.0 ± 21.6	–17.5 ± 0.4	–44.4 ± 1.1	–1.5 ± 3.4

a The data were averaged using the last 150 ns of five independent MD trajectories for each system. The energy unit is kcal/mol.

In end-point approaches for binding free energy calculation such as MM-GBSA, it is a challenge to obtain the entropy term, especially for large systems as ours. Therefore, previous study usually did not include the entropy term in binding free energy calculation assuming that this term is the same for all the ligands or peptides.^25^ In this work, we applied conformational entropy WSAS, a fast protocol for entropy calculation, for entropy estimation. WSAS has been developed and validated using a large data set including 2756 small molecules and 53 protein–ligand complexes.^32^ For the binding free energy calculation of the 53 protein–ligand complexes, WSAS performed better than normal mode analysis (NMA) which is a computational expensive method and the “default” method for entropy calculations in the AMBER package. To investigate if entropy calculation using an alternative method could change our conclusion on the impact of the phosphorylation on R2–MT binding free energy, we also estimated the entropy term using the interaction entropy method which was based on protein–ligand interaction energy.^33^ Note that the performance of the interaction entropy on a set of 15 protein–ligand complexes was also better than that of NMA.^33^ The interaction entropy energy term (*T*Δ*S*) was −31.1 kcal/mol for R2-WT–MT, −28.6 kcal/mol for R2-pSer289–MT, −27.2 kcal/mol for R2-pSer293–MT, and −26.9 kcal/mol for R2-pSer289 + pSer293–MT. The binding free energy using the interaction entropy was −53.6 kcal/mol for R2-WT–MT, −21.9 kcal/mol for R2-pSer289–MT, −28 kcal/mol for R2-pSer293–MT, and −19.0 kcal/mol for R2-pSer289 + pSer293–MT. Although these values were different from the related ones using WSAS, the order of the free binding energies of R2 peptides to MTs was unchanged. Thus, phosphorylation reduced the binding affinity of tau R2 repeat to MTs according to the MM-GBSA analysis using either WSAS or the interaction entropy method.

**Figure 3:**
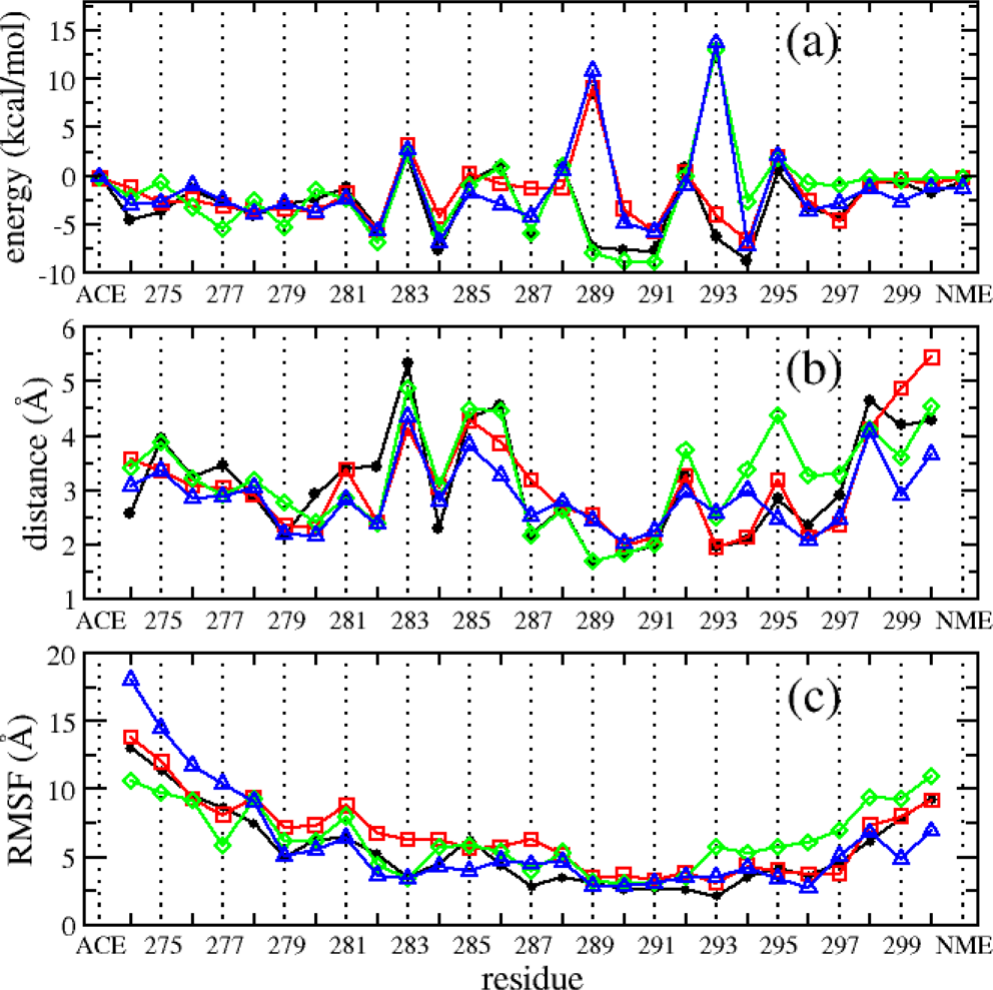
Contributions of individual residues of R2–MT binding free energy (a), the distance of R2 residues to MT tubulins (b), and mean R2 peptides’ per-residue RMSF (c). Data of R2-WT, R2-pSer289, R2-pSer293, and R2-pSer289 + pSer293 systems were illustrated by black, red, green, and blue lines and markers, respectively. The data were calculated using the last 150 ns of five individual MD trajectories for each system.

**Figure 4:**
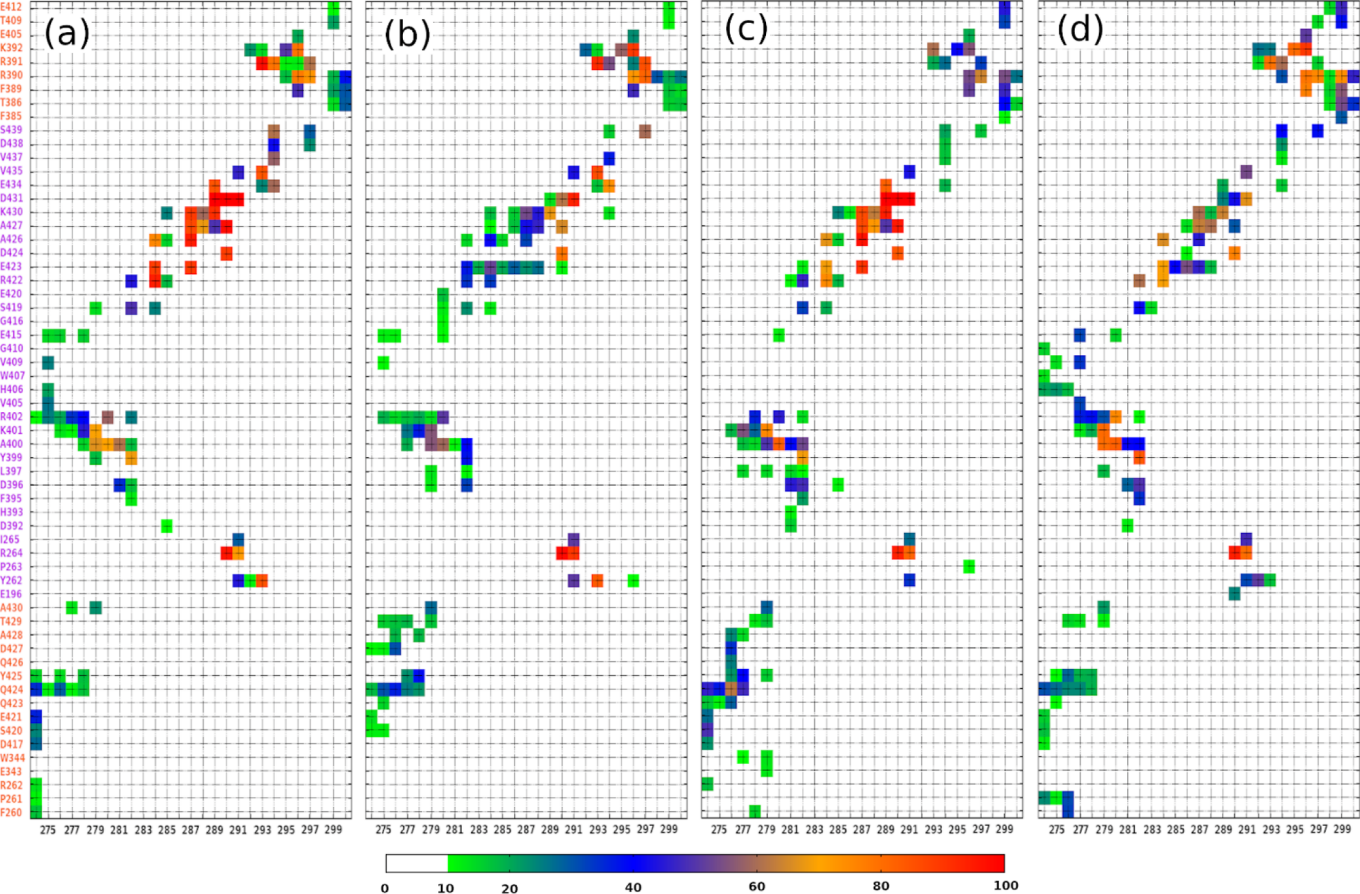
Intermolecular residue–residue interaction map of R2-WT–MT (a), R2-pSer289–MT (b), R2-pSer293–MT (c), and R2-pSer289 + pSer293–MT (d). The residues of β-tubulin were marked in orange, and the residues of α-tubulin were in purple. The data were from the last 150 ns of five trajectories for each system.

We further investigated the interaction of individual residues of R2 peptides to MT tubulins and their contribution to the peptide–protein binding energy using MM-GBSA decomposition analysis. The contribution of an individual residue of R2 peptides to the binding free energy, residue distance to MT tubulins, and per-residue root-mean-square fluctuation (RMSF) of R2 peptides for each system are shown in Figure 3. As seen, the phosphorylated residues have higher unfavorable binding energies and longer distance to MT tubulins in comparison with other ones. This result suggests that the drop of R2–MT binding affinity is mainly due to the phosphorylated residues. The binding affinity reduction of phosphorylated peptides was also reflected in the per-residue RMSF data as most residues of the phosphorylated peptides demonstrated larger RMSF than the corresponding ones in the wild-type peptide (Figure 3c). To further characterize the interaction between R2 peptides and MT tubulins, we constructed the intermolecular residue–residue interaction maps (Figure 4). The MT tubulin residues, which interacted with R2 peptides, can be classified into five groups: the MT residue group 1 includes F260, P261, R262, E343, W344, D417, S420, E421, Q423, Q424, Y425, Q426, D427, A428, T429, and A430 residues of the first MT β tubulin (β_1_) which is on the left side of Figure 1; the MT residue group 2 contains E196, Y262, P263, R264, and I265 residues of the MT α tubulin; the MT residue group 3 consists of D392, H393, F395, D396, L397, Y399, A400, K401, R402, V405, H406, W407, V409, G410, and E415 residues of the MT α tubulin; the MT residue group 4 has S419, E420, R422, E423, D424, A426, A427, K430, D431, E434, V435, V437, D438, and S439 residues of the MT α tubulin; and the MT residue group 5 includes F385, T386, F389, R390, R391, K392, E405, T409, and E412 residues of the second MT β tubulin (β_2_) which is on the right side of Figure 1. Overall, the residue–residue interaction maps of the four systems share similar patterns in the following aspects. First, they all have five interaction regions: 274–279 residues of R2 peptides and the MT residue group 1 (interaction region I); 290–293 residues of R2 peptides and the MT residue group 2 (interaction region II); 274–282 residues of R2 peptides and the MT residue group 3 (interaction region III); 280–297 residues of R2 peptides and the MT residue group 4 (interaction region IV); and 293–300 residues of R2 peptides and the MT residue group 5 (interaction region V); second, among the interaction regions, the region I had the lowest interaction frequency, while region IV showed the strongest interaction frequency during MD simulations. Third, along R2 peptides, N-terminal and C-terminal residues had low interaction frequencies (<40%), while intermediated residues (279–297) provided high interaction frequencies. This is understandable since the terminal residues usually fluctuate more vigorously than the other residues. Finally, D283 of R2 peptides weakly interacted with MT tubulins, while L282 and L284 of R2 peptides strongly bound to MT tubulins, as shown in Figures 3 and 4.

In a previous study, Jiménez investigated tau–MT binding using the same model of the R2 and MT tubulin complex (PDB Code 6CVN).^27^ He listed a set of MT tubulin residues which had strong interactions with the R2 repeat. The MT tubulin residues in his list included R262, D417, S420, E421, and Q424 of the β_1_ tubulin, R264, D396, Y399, A400, K401, R402, S419, R422, E423, A426, A427, K430, D431, E434, and V435 of the α tubulin, and F389, R390, R391, and K392 of the β_2_ tubulin.^27^ In this work, we obtained a similar result to Jiménez’s finding, except for R262, D417, S420, and E421 residues of the β_1_ tubulin, which did not show strong interactions with R2 peptides in our work. This difference may be because we capped the terminus of R2 peptides by acetyl (ACE) at the N-terminus and *N*-methylamine at the C-terminus, while Jiménez did not.

The impact of phosphorylation was clearly shown by comparing the residue–residue interaction maps of phosphorylated systems to the WT system. For the R2-pSer289–MT system, the interaction region IV, which contained pSer289 residues, had much lower interaction frequencies than that of Ser289 in the wild-type system, which strongly interacted with A427, K430, D431, and E434 residues of the α tubulin in which the frequency occupancy went up around 100% (Figure 4a), while pSer289 residue in R2-pSer289–MT only interacted with K430 and D431 residues of the α tubulin with the highest frequency occupancy about 60% (Figure 4b). For the R2-pSer293–MT system, the residues in the interaction regions II, IV, and V, which included the pSer293 residue, also gave lower interaction frequency occupancies than those ones in the wild-type system. The strong interactions between Ser293 of R2 peptides and Y262 of the β_1_ tubulin, E434 and V435 of the α tubulin, and R391 of the β_2_ tubulin in the wild-type system disappeared or significantly reduced in the R2-pSer293–MT system (Figure 4a,c). A similar picture was observed for the R2-pSer289 + pSer293–MT system, and the interaction frequency occupancy of the interaction regions including pSer289 and/or pSer293 was significantly reduced in comparison to that in the wild-type system (Figure 4a,d).

### Impact of Ser289 and Ser293 Phosphorylation on the Conformation of Monomeric Tau R2 Repeat

To study the impact of the phosphorylation on the conformation of R2 peptides themselves, we carried out MD simulations of the systems containing monomeric R2 peptides in the explicit solvent. For each monomeric system, we performed five 500 ns constant pressure and temperature (*NPT*) runs. The time courses of gyration radius (*R*_g_), SASA, and end-to-end (e2e) distance suggested that these structural parameters dramatically changed in the first 50 ns simulation time and varied around equilibrium values in the last 400 ns (Figure S2). Therefore, statistical analysis was performed using the data collected from the last 300 ns of an MD trajectory. To access the convergence of sampling, we compared the distributions of *R*_g_ and SASA in two ensemble statistics which were performed using the last 400 ns (from 100 to 500 ns) and 300 ns spanning from 100 to 400 ns (Figure S3). Obviously, the two ensemble statistics resulted in very similar distributions for the two reaction coordinates in all four systems. These results suggest that the samplings were converged, and the simulation protocol was adequate. Additionally, the analysis on the water layer (distance from the peptide to the boundary of the water box) during that last 400 ns of the simulation time showed that the sizes of water layers were greater than 20 Å most of the time (Figure S4). This result indicates that our simulation systems are large enough to exclude the aqueous volume effects that may occur when the water layer is small.^34^ In the following, we will discuss the impact of phosphorylation on the structures of the R2 peptides.

**Figure 5:**
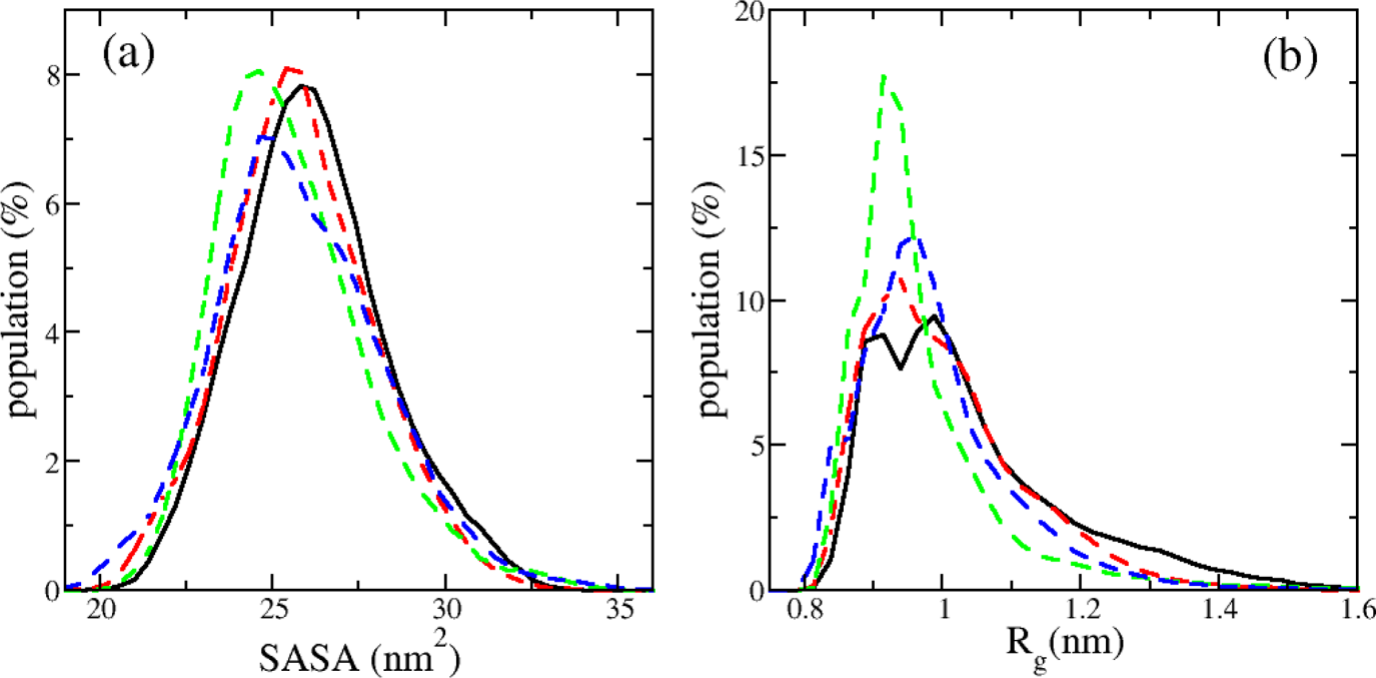
SASA and *R*_g_ distributions of monomeric R2-WT (black), R2-pSer289 (red), R2-pSer293 (green), and R2-pSer289 + pSer293 (blue) peptides. The data were collected using the last 400 ns of five independent MD trajectories for each system.

The distributions of SASA and *R*_g_ shown in Figure 5 pointed out that the phosphorylation had a little effect on the overall structures of R2 peptides, and the phosphorylated peptides were slightly more compact than the wild-type one. The averaged values of SASA were 26.17 ± 0.79, 25.81 ± 1.00, 25.57 ± 0.76, and 25.78 ± 1.14 nm^2^ for the WT, pSer289, pSer293, and pSer289 + pSer293 R2 peptides, respectively. The values of *R*_g_ are 1.04 ± 0.05, 1.00 ± 0.05, 0.97 ± 0.05, and 0.99 ± 0.05 nm for the four R2 systems correspondingly.

**Figure 6:**
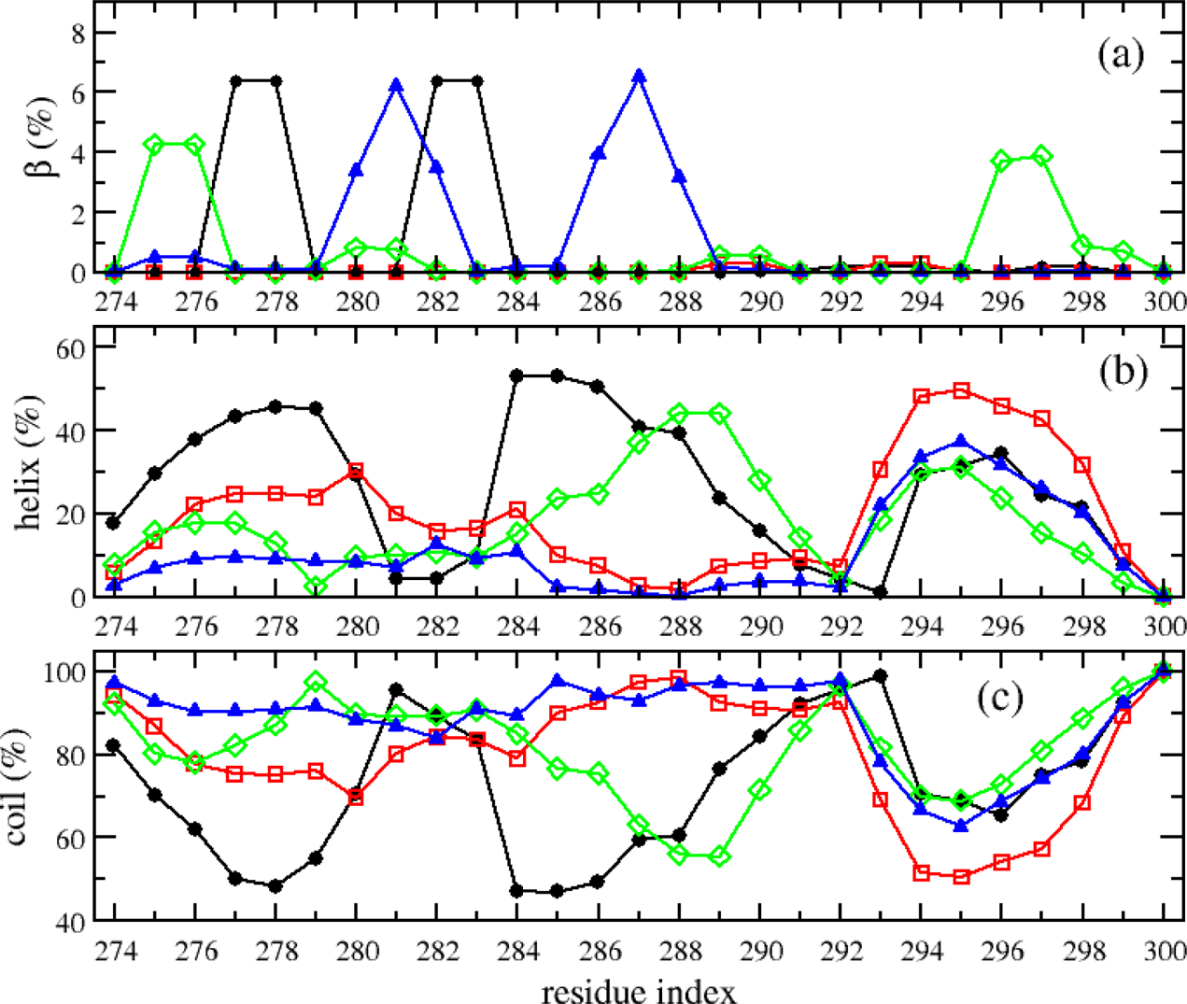
Secondary structure propensities of individual residues of monomeric R2-WT (black), R2-pSer289 (red), R2-pSer293 (green), and R2-pSer289 + pSer293 (blue) peptides. The data were collected from the last 400 ns of five independent MD trajectories for each system.

On the other hand, phosphorylation significantly changed the secondary structures of the R2 peptides. The β-contents of R2 peptides were very similar for all four systems with the averaged values around 1%, while the helix and coil contents were different among those peptides. The helix and coil contents were 26 ± 6 and 73 ± 5% for the WT peptide, 19 ± 6 and 80 ± 5% for the pSer289 peptide, 18 ± 4 and 81 ± 4% for the pSer293 peptide, and 11± and 88 ± 3% for the pSer289 + pSer293 peptide. This secondary structural result showed that the phosphorylation reduced the helix content and increased the coil content of peptides. In other words, the phosphorylation enhanced the structural transition from helix to coil, that is, from ordered to disordered, which may accelerate the aggregation of R2 peptides. The secondary structure profile along the sequence indicated that the helix–coil transition due to the phosphorylation mainly occurred at the N-terminal residues and residues in the middle of the sequence, while the C-terminal residues made little contribution (Figure 6).

**Figure 7:**
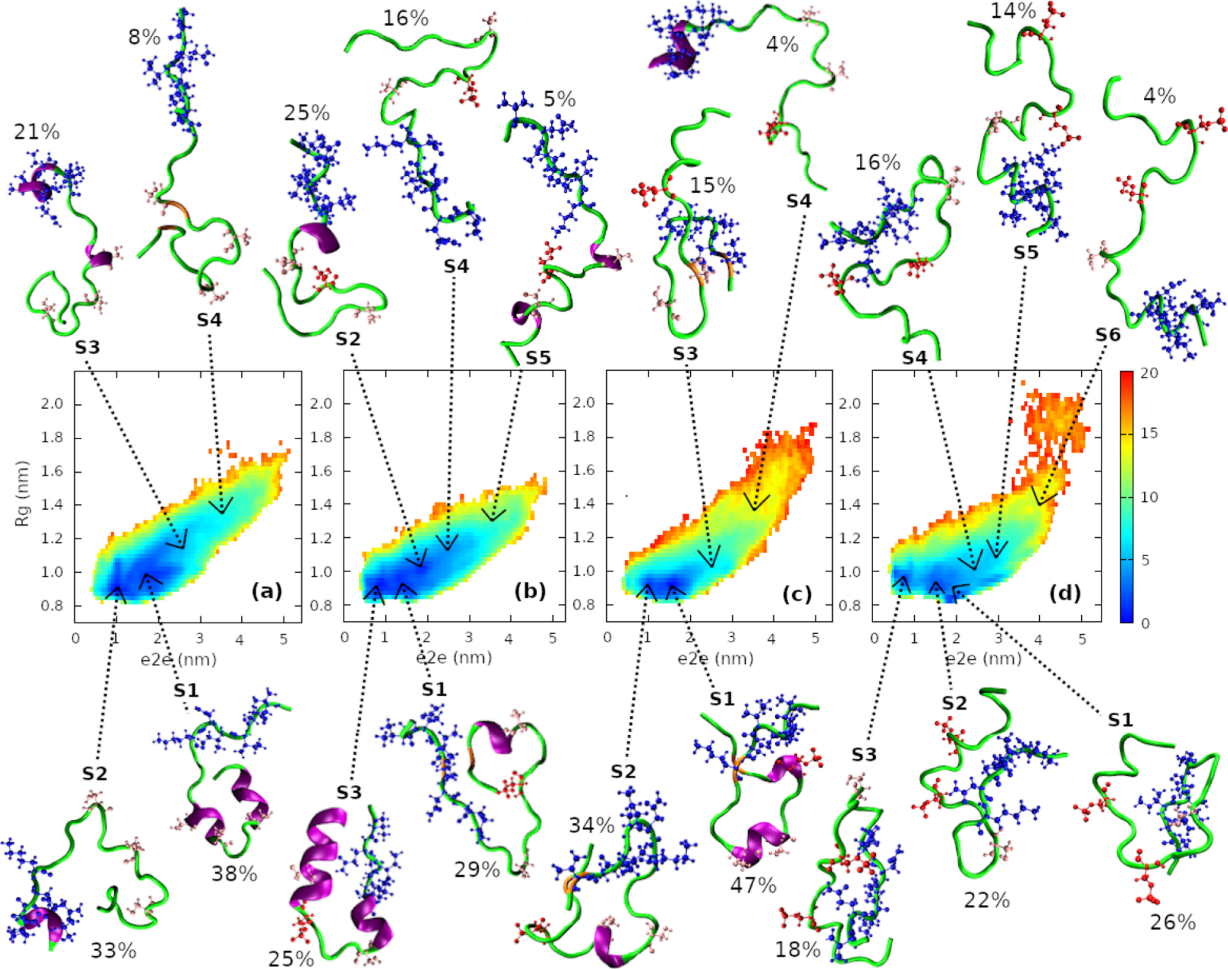
Free energy landscape of monomeric R2-WT (a), R2-pSer289 (b), R2-pSer293 (c), and R2-pSer289 + pSer293 (d) peptides. The population and representative structure of each cluster were explicitly shown. The residues of key hexapeptide PHF6* (VQIINK) were marked by blue color. The Ser and pSer residues were shown in pink and red spheres, respectively. The data were collected from the last 400 ns of five independent MD trajectories for each system.

To select representative structures of the R2 peptides, we first plotted free energy landscapes (FEL) using e2e distances and gyration radius as reaction coordinates. We then applied the *k*-means clustering method^35^ to identify the center and population of the clusters. Note that the number of clusters assigned to the *k*-means clustering analysis was estimated from the FEL plotting. An MD structure will be selected as the representative structure of a cluster if its reaction coordinate values are the closest to the cluster center. Figure 7 shows FELs of the four monomeric systems and conformational clusters with the cluster population sizes and representative structures. The numbers of clusters were 4, 5, 4, and 6 for WT, pSer289, pSer293, and pSer289 + pSer293 peptides, respectively. The helical and coil contents in the representative structures were clearly shown using schematics, and the phosphorylation-induced helix–coil transition was clearly shown. For the WT peptide, three out of four representative structures with a total of 92% of cluster population contained helical content. Particularly, two of the structures had a helix at the N-terminus where the key hexapeptide PHF6 is located. For pSer289 and pSer293 peptides, although the helical content also appeared in most of the representative structures, the total helix-containing populations, 84% for the pSer289 peptide and 85% for the pSer293 peptide, were lower than that of the WT peptide. More strikingly, no helical content showed up in any of the representative structures of the hyperphosphorylated pSer289 + pSer293 peptide, suggesting that this monomeric R2 peptide is the most disordered.

Structures of the wild-type R2 monomer have been investigated in a previous simulation study by He et al.^36^ In that work, the authors performed all-atom discrete MD simulation with the Charmm force field^37^ for protein in an implicit solvent, which is different from ours in terms of the employed force field and simulation methods. Therefore, it is interesting to compare our and He et al.’s findings. For FEL analysis, both studies found that the free energy basins of the R2 monomer were located at 0.9–1.2 and 1.0–2.5 nm with regard to *R*_g_ and e2e, respectively. In terms of secondary structures of the R2 monomer, He et al. reported the secondary structure contents of the β-sheet (4.5%), helix (9.2%), and coil (86.3%), significantly different from ours, which were 1, 26, and 73%, correspondingly. Despite the difference in secondary structure contents, helical conformations of the wild-type R2 monomer were identified in both studies, being consistent with the previous experimental observations.^38,39^

In tauopathies such as AD, abnormal phosphorylation reduces the affinity of tau to MTs, leading to tau mislocalization. The dissociated tau proteins aggregate into consequent forms including soluble oligomers, insoluble fibrils, and NFTs.^9,40−43^ These tau aggregates are toxic to the brain: NFTs are a hallmark of AD and oligomers are a major contributor in tauopathies.^41−45^ From the abovementioned tau pathology, three possible strategies were proposed to prevent tauopathies: (i) to prevent abnormal phosphorylation; (ii) to eliminate the effect of aberrant phosphorylation on tau–MT binding affinity; and (iii) to inhibit the aggregation of the phosphorylated tau proteins. All these three ways need to evaluate the impact of phosphorylation on tau–MT binding and tau aggregation. With that, one is able to identify binders to maintain the binding between phosphorylated tau and MTs and discover inhibitors to interfere with phosphorylated tau aggregation. In this work, we found that the phosphorylation at Ser289 and Ser293 sites not only reduced the binding affinity of tau repeat R2 to MTs but also promoted tau aggregation by enhancing the helix–coil structural transition of the monomeric peptide. Since repeat R2 plays an important role in both MT binding and aggregation of full-length tau protein, we suggest that the phosphorylation is likely to have a similar impact on the full-length tau as on repeat R2. Thus, further studies on the impact of the phosphorylation using full-length tau proteins and the role of phosphorylation in tauopathies are of great interest. We also suggest that phosphorylation at the two repeat R2 serine residues must be taken into account in drug development for tau-related diseases.

## Conclusions

In conclusion, we first investigated the effects of abnormal Ser289, Ser293, and Ser289/Se293 phosphorylation on the free energy of tau R2–MT binding. We showed that phosphorylation at Ser289 and Ser293 significantly reduced the interactions between the tau R2 peptide and MT tubulins, with most contribution from the modified residues themselves and some surrounding residues at the sites of phosphorylation. We then characterized the conformational ensembles of four monomeric tau R2 peptides, and a helix to coil structural transition was discovered when Ser289 or Ser293 and both serine residues were phosphorylated. This from ordered to disordered structural transition may be a driving force of tau aggregation. For the first time, we systematically studied the pathological impact of the phosphorylation on MT binding and conformation of tau repeat R2 using the state of the art of all-atom MD simulations. Considering the vital role of the repeat R2 on MT binding and aggregation of full-length tau proteins, the phosphorylation at Ser289 and Ser293 sites is likely to play an important pathological role in AD pathogenesis. Therefore, this work can promote further investigations on the role of phosphorylation in AD pathogenesis by both the simulation and experimental means.

## Materials and Methods

### System Design

For the R2 and MT complex systems, the experimental structure of the R2 and MT tubulin complex (PDB code 6CVN)^27^ was used as an initial structure of the wild-type R2–MT complex. The initial structures of phosphorylated R2–MT complexes were obtained by performing mutations from Ser to pSer at the corresponding residues of R2-WT. Each complex was placed at the center of an octagonal box with a box size of 14.7 nm, and the box was then solvated by explicit TIP3P water. Sodium and chloride ions were added to the solvated system to neutralized the system and to maintain ∼0.15 M salt concentration. The smallest distance between any atom of the complex and the box border was 10–12 Å. The system contains about 69,000 water molecules. For the monomeric peptide systems, the initial structure of R2-WT was also taken from the experimental structure of the R2–MT complex. Similar protocols were used to generate phosphorylated R2 and to solvate the peptide in explicit water. Each monomer system has 8.5 nm box size, containing 14,400 water molecules and 0.15 M salt, and the whole system was neutralized.

### Simulation Details

The pmemd.cuda module of the AMBER 18 software package^46^ was used to perform all MD simulations. The ff14SB force field.^47^ was used to model peptides/protein, and the TIP3P water model^48^ was used to represent the explicit solvent. The parameters for a phosphorylated residue were taken from Homeyer et al.’s work.^49^ The periodic boundary conditions were applied for simulations to make a system more like an infinite system and to improve the rigor and realism of the molecular model. The long-range Coulomb interaction was evaluated by means of the particle-mesh Ewald method^50^ with a cutoff of 1.0 nm. The van der Waals interactions were calculated by means of 1.0 nm atom-based nonbonded lists, with continuous corrections applied to the long-range parts. The constant pressure simulations were carried out at 1 atm via the Berendsen barostat^51^ with pressure relaxation time τ_p_ = 3.0 ps. Each system underwent the following steps. First, the steepest descent minimization followed by a conjugate gradient minimization with the peptide atoms fixed at their initial positions; then, the steepest descent minimization followed by conjugate gradient minimization was carried out without applying constraint to any atoms. The minimization stage was followed by a short constant volume MD simulation, while the system was heated from 0 to 310 K with weak restraints on the protein atoms. Next, a Langevin dynamics at constant temperature (310 K) and constant pressure (1 atm) was applied for 100 ps, after which the density of the system was found to be stable around 1.0 g/cm^3^. Finally, in the sampling phase, a constant volume MD run at 310 K was generated using the leap-frog algorithm with a time step of 2 fs. The temperature was regulated using Langevin dynamics with a collision frequency of 1 ps^–1^. The SHAKE algorithm^52^ was applied to all bonds involving hydrogen atoms. Conformations were saved every 10 ps for post-analysis. For the complex systems, five 200 ns *NPT* runs were carried out for each system. For the monomeric systems, five 500 ns *NPT* runs were carried out for each system. The snapshots were saved every 100 ps of the simulations for post-analysis.

### Data Analysis

#### Binding Free Energy

The binding free energy of R2 peptides to MT tubulins was calculated using MM-GBSA–WSAS.^28,29^ The binding free energy was defined as follows: Δ*G*_bind_ = Δ*G*_complex_ – (Δ*G*_MT_ + Δ*G*_peptide_), where Δ*G*_complex_, Δ*G*_MT_, and Δ*G*_peptide_ are the free energies of the complex, MT tubulins, and R2 peptide, respectively. The total free energy was estimated by summing up the contributions of different energy terms according to the following equation: Δ*G*_bind_ = Δ*E*_int_ + Δ*E*_vdw_ + Δ*E*_eel_ + Δ*G*_p_^sol^ + Δ*G*_np_^sol^ – *T*Δ*S*. Δ*E*_int_ stands for the internal energy change, which is canceled out when applying the “single trajectory” sampling protocol as we carried out in previous study;^50,53^ Δ*E*_vdw_ and Δ*E*_eel_ are the van der Waals and gas-phase electrostatic energies, respectively; Δ*G*_p_^sol^ and Δ*G*_np_^sol^ are the polar and nonpolar components of the solvation free energy, respectively. *T* is the absolute temperature; Δ*S* is the change in the conformational entropy calculated using the WSAS method.^32^

#### Secondary Structure Contents

The secondary structure contents classified into β, helix, and random coil families were calculated using the STRIDE algorithm.^54,55^ Here, the helix content includes 3–10 helices, π helix and α helix, the β one consists of extended residues, and the rest belongs to random coil. CPPTRAJ tools^56^ were used to calculate the SASA, gyrate (*R*_g_), and distances. The LCPO algorithm^57^ was applied for the SASA calculation. The intermolecular residue–residue interaction map was constructed using 0.3 nm cutoff for the distance between a residue pair.

#### Free Energy Landscape

The free energy surface along the N-dimensional reaction-coordinated  is given by , where *P*(*V*) is the probability distribution of MD data represented by histograms. *P*_max_ is the maximum of a distribution, which is subtracted to ensure that the free energy minimum has a Δ*G* of 0. The *k*_B_ and *T* are Boltzmann constant and simulation temperature, respectively. In this study, we used e2e distances and gyration radius of the peptide as reaction coordinates to construct the two-dimensional FEL.

## Supporting Information Available

Time courses of helix content, coil content, SASA, and RMSD of R2 peptides in the R2–MT complex systems; time courses of gyration radius, SASA, and e2e distance of monomeric R2 peptides; distributions of gyration radius, SASA, and e2e distance of monomeric R2 peptides; and distribution of the distance between R2 peptides and the water box boundary in different monomeric systems (PDF)
