# Supplementary material for: Phosphorylation of Tau R2 Repeat Destabilizes Its Binding to Microtubules: A Molecular Dynamics Simulation Study
Source: ACS Chem Neurosci. 2023 Jan 20;14(3):458–67. doi: 10.1021/acschemneuro.2c00611 (PMC10032563; doi:10.1021/acschemneuro.2c00611)
Supplement: Supplementary file 1 — cn2c00611_si_001.pdf [file cn2c00611_si_001.pdf]

Phosphorylation of Tau R2 Repeat Destabilizes its Binding to Microtubules: A Molecular Dynamics Simulation Study

SUPPORTING INFORMATION

Viet Hoang Man,[1]* Xibing He,[1] Jie Gao,[2] and Junmei Wang[1]*

[1] *Department of Pharmaceutical Sciences and Computational Chemical Genomics Screening Center, School of Pharmacy, University of Pittsburgh, Pittsburgh, PA 15261, USA.*

[2] *Department of Neuroscience, The Ohio State University Wexner Medical Center, Columbus, OH 43210, USA.*

E-Mails:

Viet Hoang Man: vhm3@pitt.edu
Xibing He: xibing.he@pitt.edu
Jie Gao: Jie.Gao@osumc.edu
Junmei Wang: junmei.wang@pitt.edu

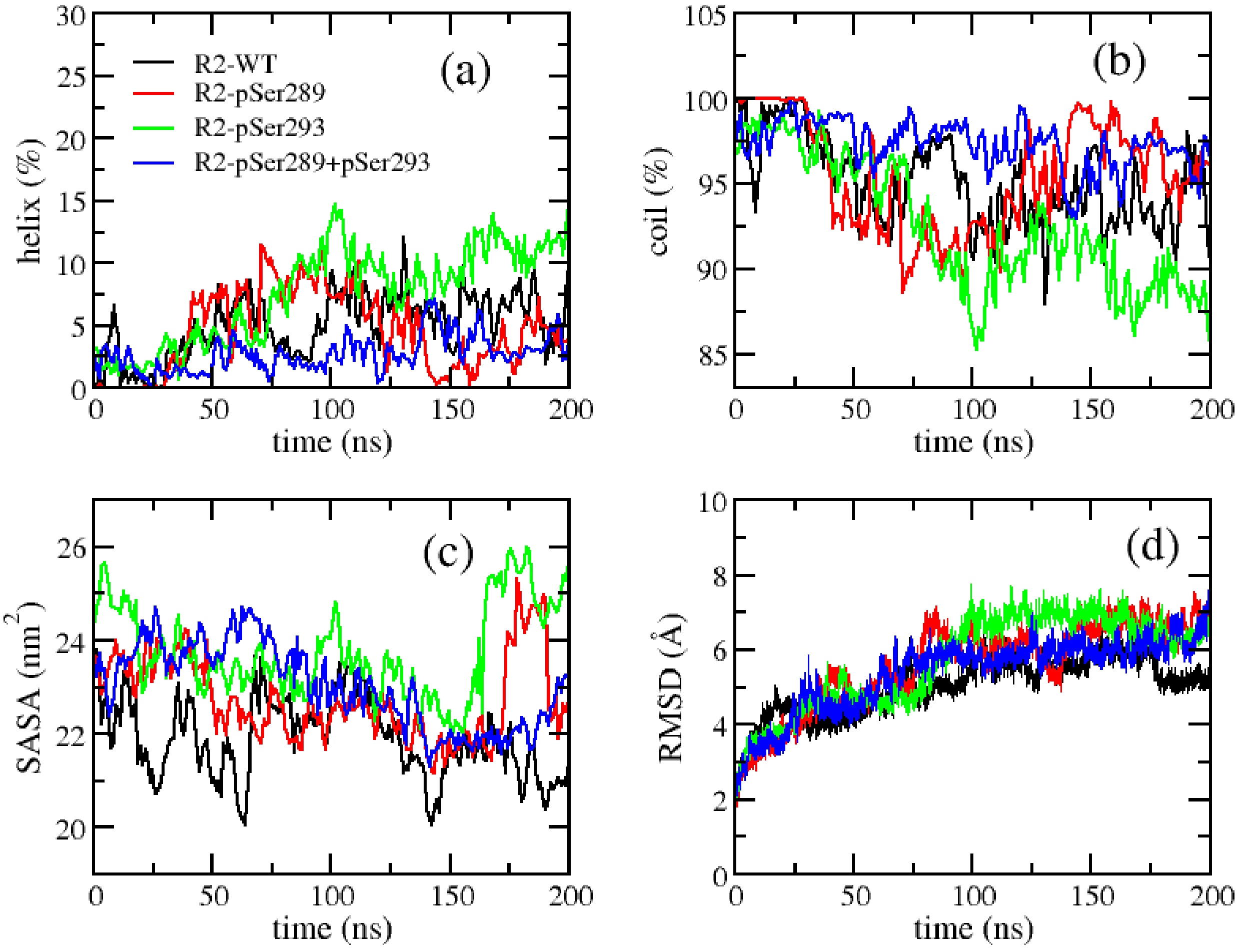

Figure S1: Time courses of helix content (a), coil content (b), solvent accessible surface area (c), and RMSD (d) of R2 peptides in the R2-MT complex systems. The data was averaged over five independent MD trajectories for each system.

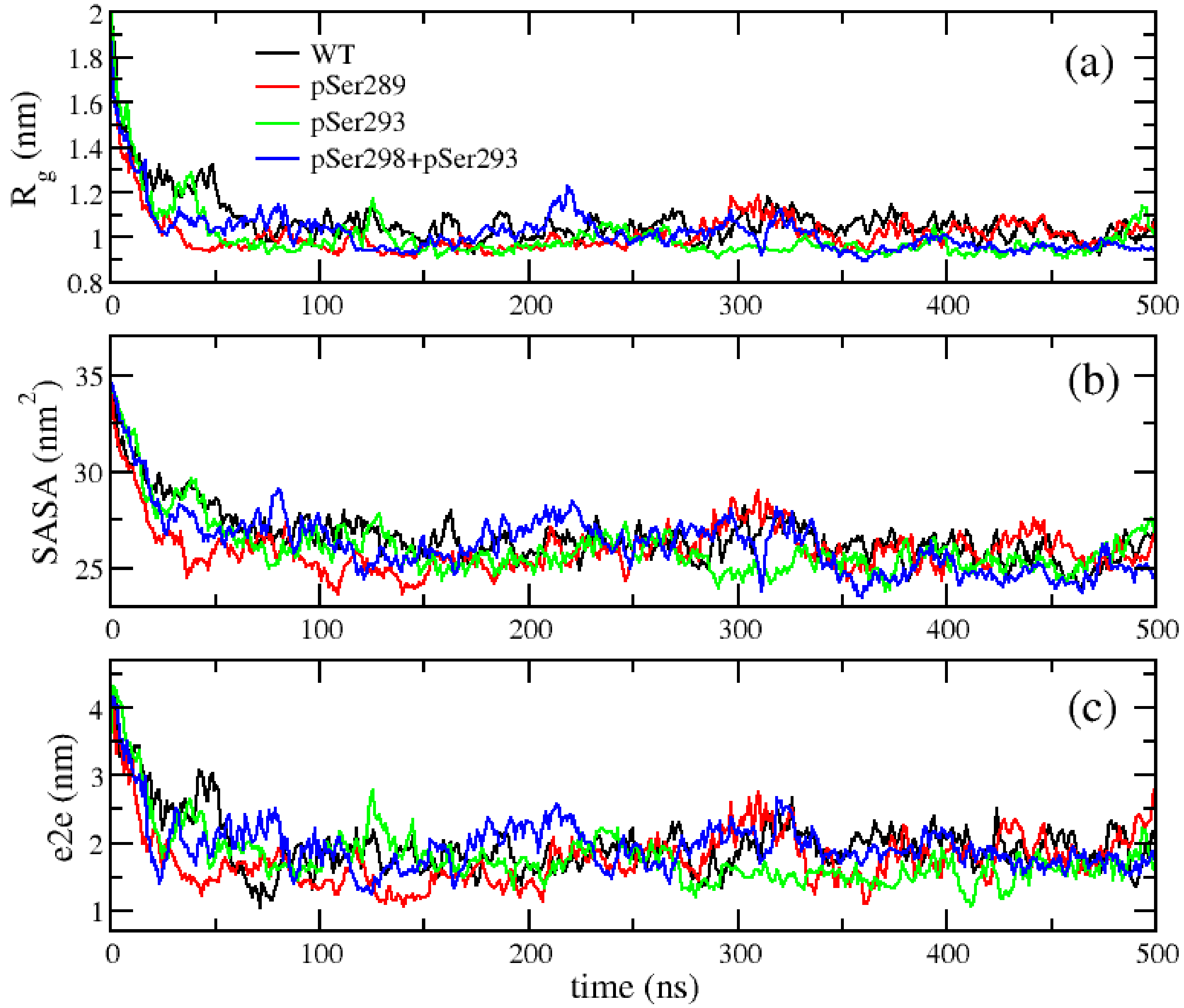

Figure S2: Time courses of gyration radius (a), solvent accessible surface area (b) and end-to-end distance (c) of monomeric R2 peptides. The data were averaged from five independent MD trajectories for each system.

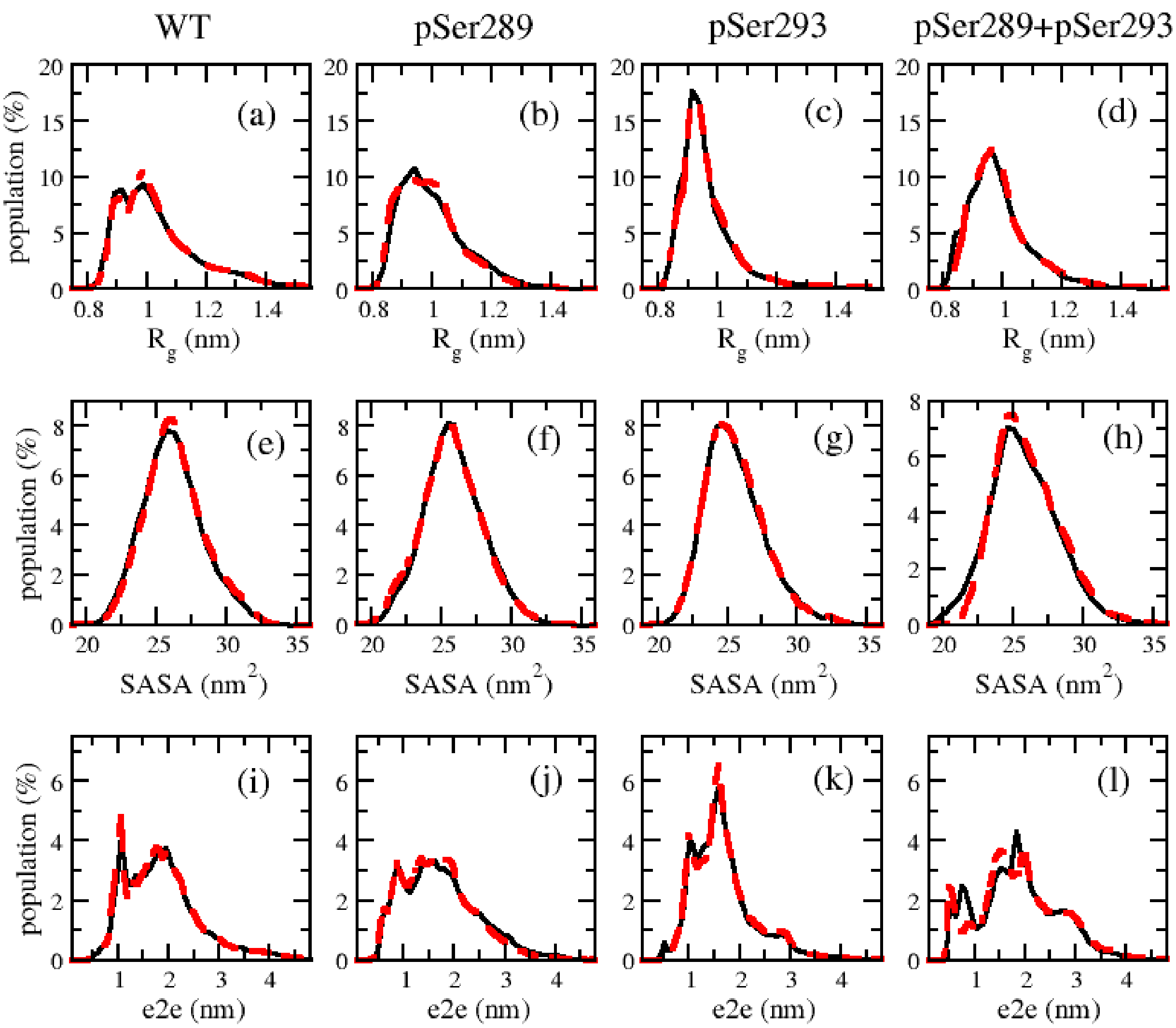

Figure S3: The distributions of gyration radius, solvent accessible surface area, and end-to-end distance of monomeric R2 peptides. Two ensemble statistics were conducted for comparison purpose, one using all the last 400 ns (from 100 ns to 500 ns) of all five trajectories (black lines), and the other using 300 ns spanning from 100 ns to 400 ns of all five trajectories (red dashed lines).

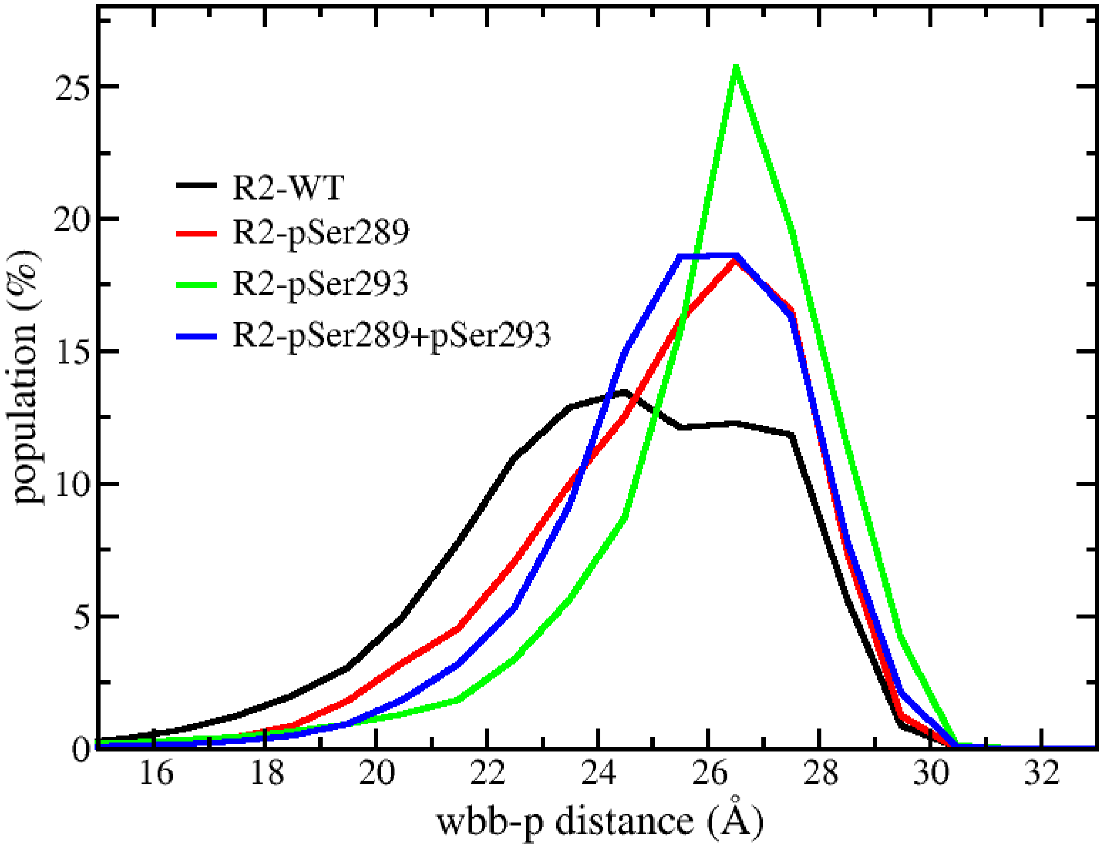

Figure S4: Distribution of the distance between R2 peptides and water box bounder in different monomeric systems.
